# Development of a Method for Peeling Off Paper from Celluloid Pictures for Animation Films

**DOI:** 10.3390/polym15030690

**Published:** 2023-01-30

**Authors:** Masahiro Kaneko, Joon Yang Kim, Minori Ishida, Mika Kawai, Tetsu Mitsumata

**Affiliations:** 1 Graduate School of Science and Technology, Niigata University, Niigata 950-2181, Japan; 2 Graduate School of Modern Society and Culture, Niigata University, Niigata 950-2181, Japan

**Keywords:** cellulose acetate, paper, acrylic emulsion, celluloid picture, paint, animation

## Abstract

During the storage of celluloid pictures for animation films over half a century, an interleave paper adhered to acrylic paint. The purpose of this study is to establish a methodology to cleanly remove the paper from the paint. A layered film, a replica of the celluloid pictures, adhered with paper was prepared and immersed in water or ethanol. The effect of these solvents on the peeling behavior was investigated using a peel test. The maximum peel force for the dry layered film in was distributed at ~0.5 N, independently of the peel speed. The peel force was significantly reduced after the layered film was immersed in pure water or ethanol. A morphological observation revealed that the dry paper was peeled off via the cohesive failure of the paper. After the layered film was immersed in pure water, the paper was also peeled off via cohesive failure. The layered film immersed in ethanol was peeled off at the paper/paint interface. To clear the effect of the volume change in the paint on peel behavior, the relative volume was determined via image analysis. The relative volume of paint was 1.56 in pure water and 1.37 in ethanol. It can be considered that the large difference in the volume of paint induces a large shear stress at the paint/paper interface.

## 1. Introduction

Many animation films have been produced since the beginning of the 20th century in Japan, and both Manga and animation are popular. Animation is one of the most important cultural aspects of Japan [1]. In the 1960s, the number of animations produced increased dramatically, with approximately 100 films produced in one year. Nowadays, animation is mainly made from digital images; however, prior to the 1980s, it was mainly made from a transparent film called celluloid picture, upon which pictures were drawn. An animation cell is a transparent polymer film upon which pictures are painted with acrylic emulsion paints (acrylic watercolors). Normally, the movement of the animation is produced using several celluloid pictures (still pictures) per second. In recent years, the artistic value of animation cells has become extremely high, and efforts to preserve them as objects of cultural heritage have significantly increased [2,3,4,5].

In addition to the preservation of celluloid pictures, research is also conducted on the constituent of polymers. Needless to say, the chemical composition of a celluloid picture is important for preservation in good conditions. Giachet et al. reported that cellulose diacetate and cellulose triacetate used as polymer films for celluloid pictures were identified by TGA and FTIR measurements. They demonstrated that the polymer used in celluloid pictures was transferred from cellulose diacetate to cellulose triacetate during the period of 1981–1983 [3]. Giachet et al. also investigated the evolution of plasticizer used for cellulose diacetate and cellulose triacetate [6]. Beltran et al. revealed that the adhesion between celluloid pictures and gum-arabic-based paint is strongly enhanced in high-humidity conditions, resulting in the damage of celluloid pictures [7]. Another serious problem we focused on is the adhesion between celluloid picture and paper, which is an interleave material between the celluloid pictures. Celluloid pictures were stacked and stored on interleave paper for half a century. The interleave paper is to prevent the paper from adhering to the polymer film; however, during storage, the interleave paper unfortunately adhered to the celluloid picture. It is important to cleanly peel off the interleave paper from celluloid picture with high artistic values for their preservation, which has not been investigated thus far.

It is empirically known that paper exhibits cohesive failure and is not peeled at the paper/paint interface. Most of the paints used in animation in the period of 1970–1990, called animation colors, were acrylic emulsion paints. The paint is an emulsion of micron-order acrylic resins dispersed in water, which become water-resistant after drying. This is a feature that prevents the painted picture from blotting out with water. Thus, in order to cleanly remove the interleave paper from celluloid pictures, it is necessary to increase the strength of the paper itself and/or to reduce the adhesive strength of the paper/paint interface.

As celluloid pictures are quite precious, the picture used in the animation films cannot be used for the peel test. In this study, we prepared a replica of a celluloid picture, which is a layered film of paper, acrylic emulsion paint, and cellulose acetate. The peel force of the paper from the layered film was measured using a 90° peel test. To elucidate the effect of volume changes in the solvents of the materials constituting the layered film on the peel behavior, volume changes were determined using image analysis. The absorption ratio and the relative volume of the layered film immersed in pure water and ethanol were measured to discuss the mechanism of the peel behavior. In addition, the peel surface for the layered film was observed using a digital scope to investigate the optimum conditions for cleanly peeling the paper from the paint. The goal of this study is to find the optimal solvent by which the interleave paper can be removed from the celluloid pictures.

## 2. Experimental Procedures

### 2.1. Preparation of Paper/Paint/Cellulose Acetate Layered Film

A water-based red paint made from a poly(methyl methacrylate) emulsion (1459, Sun Note Co., Ltd., Osaka, Japan) was applied on the cellulose acetate film (CA film, Loppo, LLC., Tokyo, Japan). The CA film painted was dried in air for 1.5 min, reaching a water content of approximately 40%. Then, a paper (R100, Japan Pulp & Paper Co., Ltd., Tokyo, Japan) was put on the paint to obtain a layered film. A weight of 1.5 kg, which is nearly equal to the weight of cells stacked 50 cm high, was loaded on the top of the layered film for 5 min. The layered film was then dried on a hot stage at 100 °C for 120 min to remove residual water in the paint. In order to ensure the adhesion conditions are uniform, samples were produced once under ranges of humidity (30–35% RH) and temperature (20–24 °C).

### 2.2. Immersion of the Layered Film in Solvents

The layered films were immersed in pure water or ethanol for 30 min at room temperature. Then, after the immersion, the films were provided for the peel test.

### 2.3. Peel Test

**Figure 1:**
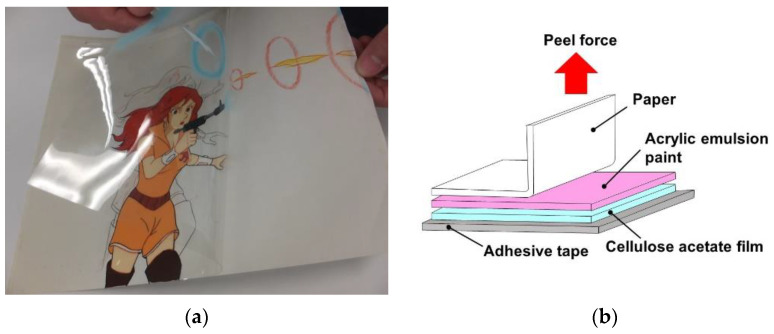
(**a**) An example of actual animation cell adhered to paper stored over 40 years. (**b**) Schematic illustration for the peel test carried out in this study. The peel force was measured when a paper was peeled off from the layered film.

The 90° peel test of the layered films was performed using a universal tensile tester (EZ-Test EZ-SX, Shimadzu Co., Kyoto, Japan), as shown in Figure 1. The layered film was adhered using an adhesive tape (SKB-20R, 3M Japan Ltd., Tokyo, Japan) on the sample stage of the tensile tester. The peel distance was 10 mm, and the peel speeds were 1, 10 and 100 mm/min. The data were determined from averages of three different samples, as shown in the figure.

### 2.4. Observation of Peeled Surface

The peeled surface of the layered film was observed using a digital scope (1080P, Linkmicro, Henan, China).

### 2.5. Absorption Ratio and Relative Volume

The paint described in 3.1 was dried on a hot stage at 100 °C for 6 h. The dried paint with a disk shape (20 mm in diameter, 1 mm thick) was immersed in pure water or ethanol at room temperature for 30 min and then weighed. The absorption ratio of paper, paint, and CA film for these solvents was defined as the change in weight before and after immersing the solvents dividing by the dry weight. The relative volume, which is the volume ratio before and after immersing the solvent, was determined from the size of samples using image analysis. The data were determined from averages of three different samples, as shown in the figure.

## 3. Results and Discussion

**Figure 2:**
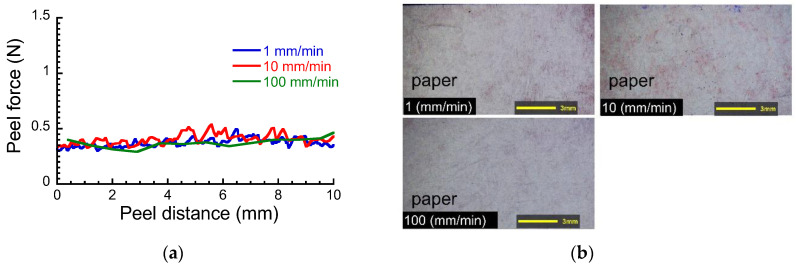
(**a**) Peel force vs. peel distance and (**b**) the peeled surface at various peel speeds for the layered films in dry.

Figure 2a exhibits the peel force against the peel distance at various peel speeds when the paper was peeled off from the layered film in dry. The peel force fluctuated around 0.4 N and is independent of the peel speed. Figure 2b demonstrates the photographs of a peeled surface at various peel speeds for the layered films in dry conditions. It can be seen that most of paper fibrils remained on the paint. This indicates that the paper was peeled off due to the cohesive failure of paper. Therefore, it can be considered that the cohesion force between paper fibrils is lower than the adhesion force between the interfaces of paper/paint, paint/CA film, or the cohesion force of the paint.

**Figure 3:**
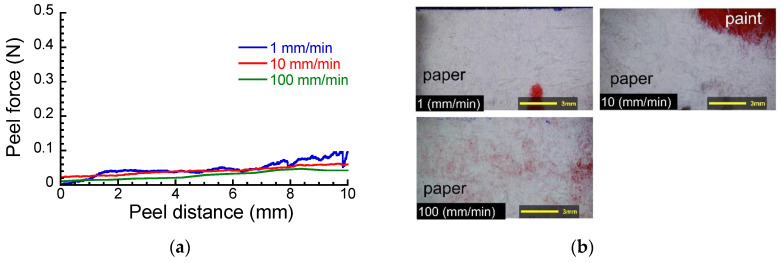
(**a**) Peel force vs. peel distance and (**b**) the peeled surface at various peel speeds for the layered films after immersed in pure water.

Figure 3a shows the peel force against the peel distance at various peel speeds when the paper was peeled off from the layered film after immersed in pure water. The peel force fluctuated less compared to the dry sample, and it was significantly reduced by immersing in pure water at all peel speeds. The photographs of a peeled surface at various peel speeds for the layered film after immersion in pure water are shown in Figure 3b. Many paper fibrils remained on the paint although the amount of the fibrils was less than that for the dry sample. This means that the peeled surface is unchanged, although the peel force was significantly reduced following immersion in pure water, i.e., the peel was caused by the cohesive failure of paper. Therefore, it can be considered that the large decrease in the peel force was caused by the decrease in the cohesion force between paper fibrils, mainly arising from the entanglement of the fibrils.

**Figure 4:**
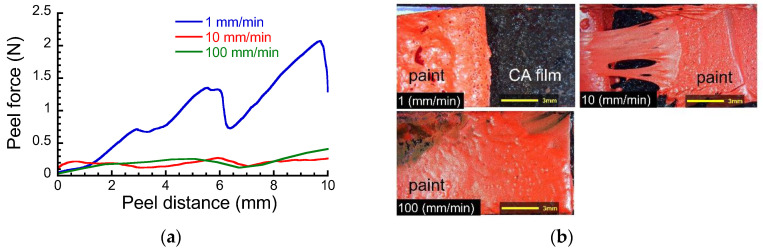
(**a**) Peel force vs. peel distance and (**b**) the peeled surface at various peel speeds for the layered films after immersion in ethanol.

Figure 4a depicts the peel force against the peel distance at various peel speeds, when the paper was peeled off from the layered film after immersed in ethanol. The peel force demonstrated less fluctuation and was higher than that for samples immersed in pure water. Particularly, the peel force at 1 mm/min showed the highest value for all samples, suggesting the mechanism of peeling is different from others. Figure 4b demonstrates the photographs of a peeled surface at various peel speeds for the layered film after immersion in ethanol. At 1 mm/min, the paper was peeled off at the paint/CA film interface, suggesting that the adhesion force between paint and CA film was reduced by immersing in ethanol. On the other hand, at 10 and 100 mm/min, the paper was peeled off at the paper/paint interface. Therefore, the immersion in ethanol weakens the adhesion force of both paper/paint and paint/CA film interfaces. In addition, it was also found that the adhesion property at the paper/paint interface depends on the peel speed, which is likely due to the viscoelastic property of paint swollen by ethanol. From an application point of view, the obtained results reveal that there is a risk of damaging celluloid pictures when the peeling speed is low.

**Figure 5:**
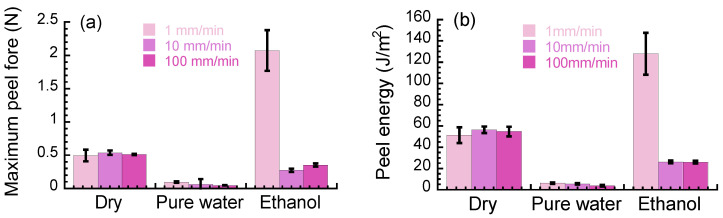
(**a**) Averaged peel force and (**b**) peel energy at various peel speeds for peeling off the paper from the layered film.

Here, the effect of solvents on the peeling behavior mentioned above is summarized. Figure 5a shows the maximum peel force at various peel speeds when the paper was peeled off from the layered film. The maximum peel force for dry samples was approximately 0.5 N, independent of the peel speed. Samples immersed in pure water showed low values of the maximum peel force that slightly decreased with peel speed. The maximum peel force for samples immersed in ethanol took the highest value at 1 mm/min and those at 10 and 100 mm/min were higher than the values for samples immersed in pure water. Figure 5b exhibits the average peel energy at various peel speeds when the paper was peeled off from the layered film. The peel energy was determined by integrating the peel force with the peel distance. The peel energy for the layered film in dry conditions was independent of the peel speed and was distributed around 50 J/m^2^. The peel energy for the layered film immersed in pure water showed extremely low values (~10 J/m^2^) and a decreasing trend with peel speed. On the other hand, the peel energy for the layered film immersed in ethanol was highest at 1 mm/min (~130 J/m^2^), and it was around 20 J/m^2^ at 10 and 100 mm/min.

**Figure 6:**
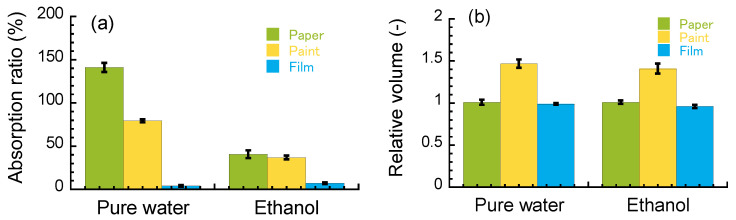
(**a**) Absorption ratio of the solvents and (**b**) the relative volume for the layered films immersed in pure water or ethanol for 30 min.

To clear the effect of the volume change for the paint on the peel behavior, the relative volume was determined by image analysis. There are many studies on the water-absorption behavior of paper [8,9,10], and the water absorption of acrylic paints is also well known [11,12]. Figure 6a shows the absorption ratio of pure water and ethanol for the paper, paint, and CA film used in the layered film. The absorption ratio of paper in pure water and ethanol was 157% and 49%, respectively. On the other hand, the absorption ratio of paint in pure water and ethanol was 62% and 35%, respectively. The absorption ratio of CA film in pure water and ethanol was 3.6% and 6.4%, respectively. The absorption ratio decreased in the order of paper, paint, and CA film, both in pure water and ethanol. Figure 6b shows the relative volume of the paper, paint, and CA film used in the layered film. The relative volumes of the paper in pure water and ethanol were 0.95 and 0.98, respectively, indicating that there are no clear changes in volume for both pure water and ethanol. Accordingly, it is suggested that the paper absorbs water due to capillary force via pores made of paper fibrils [13]. The relative volumes of the CA film in pure water and ethanol were 0.99 and 0.94, respectively. Meanwhile, the relative volumes of the paint in pure water and ethanol were 1.6 and 1.4, respectively, suggesting that the paint was swollen in both pure water and ethanol. Therefore, a large difference in the volume change occurs at paper/paint or paint/CA film interfaces. The fact that the dried paint was not dissolved in these solvents strongly indicates that the acrylic emulsion formed a physical gel after the paint dried, resulting in a swelling behavior characterized by cross-linked polymer networks. When hypothesizing isotropic volume change, this volume change causes a strain of 11% for each direction. Therefore, a large difference in the volume change occurred at the paper/paint and/or paint/CA film interfaces when the layered film was immersed in pure water or ethanol. A strong shear force should be induced at the interface due to a large difference in the expansion ratio. As mentioned above, the paper immersed in pure water demonstrated cohesive failure, rather than interfacial detachment at the paper/paint interface. The interaction between paper fibrils is reduced, and thus the paper absorbed a large amount of water, resulting in cohesive failure. Conversely, it is suggested that the interaction between paper fibrils was not reduced, since the paper did not absorb a large amount of ethanol, which increases its the mechanical strength of the paper.

Finally, we briefly describe a possible discrepancy between the replica films prepared in this study and the actual films stored for half a century. The layered film prepared in this experiment was solidified in a short time (~3 h). Therefore, it can be considered that the gel network of an acrylic emulsion is solidified in unstable state. The actual celluloid pictures solidified over a much longer time period. In the liquid state before solidification, the acrylic emulsions that form the gel network can move in the paint. This means that the resultant structure of a gel network is considered to be different between the current experiment and actual celluloid pictures. Although actual celluloid pictures can only be tested using non-destructive tests, this kind of aging effect should also be further studied.

## 4. Conclusions

The peel behavior of paper peeled off from a layered film consisting of paper, paint, and cellulose acetate film was investigated, and the effect of solvents on peel behavior was evaluated. An interesting behavior was observed for the layered film immersed in ethanol; the paper was peeled off at the paper/paint interface. This strongly indicates that the interaction between the paper and paint can be reduced by immersing the layered film in ethanol. This is roughly explained by the differences in solvent absorption and the volume changes in neighboring layers because the large difference in the expansion ratio should induce a strong shear force at the interface. Thus, it can be found that the peel interface is strongly affected by the solvent absorption or volume changes of each layer via immersion in solvents. The findings obtained in this study can help to cleanly peel off the paper adhered to celluloid pictures. This study not only contributes to the preservation of celluloid pictures for animation with high historical and cultural value, but also brings economic benefits via the repairing of pictures with a high market value.
